# House dust mite subcutaneous immunotherapy has sustained long‐term effectiveness on allergic rhinitis and asthma: A 10‐year follow‐up

**DOI:** 10.1002/iid3.1004

**Published:** 2023-10-13

**Authors:** Elena Rodriguez‐Plata, Ariel Callero Viera, Monica Ruiz‐Garcia, Aida Gomez‐Cardenosa, Eva Nieto, Jose Carlos García‐Robaina

**Affiliations:** ^1^ Allergy Department Hospital Universitario Nuestra Señora de Candelaria Santa Cruz de Tenerife Spain; ^2^ Medical Affairs and Clinical Department LETI Pharma Madrid Spain

**Keywords:** allergic asthma, allergic rhinitis, house dust mite, long‐term effectiveness, subcutaneous immunotherapy

## Abstract

**Background:**

Maintenance doses for allergen immunotherapy (AIT) have been recommended for at least 3 years but little data on long‐term efficacy is available depending on AIT duration. To show sustained efficacy 10 years after completion of treatment with depigmented‐polymerized house dust mite (dpg‐pol HDM) allergen extract in adults with asthma and/or rhinoconjunctivitis.

**Methods:**

Patients included in a double‐blind placebo‐controlled AIT study with dpg‐pol HDM allergen extract were reviewed at completion of the perennial treatment and 10‐year follow‐up (10y‐FU). Change in symptom and rescue medication score was the primary objective. Visual analog scale (VAS), asthma control test (ACT), and degree of disease control were the secondary objectives. A comparative analysis between patients who underwent AIT treatment for <3 years and ≥3 years was performed.

**Results:**

Data from 31 patients (mean age 38 years) were available at 10y‐FU. All had asthma and 29 had rhinoconjunctivitis at baseline. Twenty‐three patients were treated ≥3 years and 8 for <3 years. Seventeen (55%) patients were asymptomatic at completion of AIT, with significant differences for nasal, conjunctival, and bronchial symptoms (*p* < .0001) compared with baseline only in those patients treated ≥3 years. Nine (52.9%) patients remained completely asymptomatic at 10y‐FU, all were treated for ≥3 years. Moreover, significant reduction in the number of patients with rhinitis (*p* = .0117), conjunctivitis (*p* < .0001), and bronchial (*p* = .0005) symptoms was observed at 10y‐FU compared with baseline only in the ≥3 years treated. Ten (32.3%) patients did not require any rescue medication at 10y‐FU, all had been treated for ≥3 years. ACT at 10y‐FU showed a good control of asthma (median 23.5; 95% IC[22.0, 25.0]). No significant differences were observed between VAS at end of treatment compared with VAS at 10y‐FU.

**Conclusions:**

Sustained clinical efficacy is achieved 10 years after completion of depigmented‐polymerized HDM, however, these findings were observed only if patients are treated for at least 3 years.

## Key Messages

- A minimum of 3 years AIT treatment is required to obtain a sustained long‐term effectiveness.
- Dpg‐pol HDM is able to remove allergic rhinitis and asthma symptoms completely.
- Three‐year treatment with dpg‐pol HDM is able to maintain effectiveness 10 years after treatment.

## Graphical abstract

Patients included in a double‐blind placebo‐controlled AIT study with dpg‐pol HDM allergen extract were reviewed at completion of the perennial treatment and 10‐year follow‐up (10y‐FU). Sustained clinical efficacy is achieved 10 years after completion of depigmented‐polymerized HDM, however, these findings were observed only if patients are treated for at least 3 years.

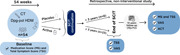

## 1 INTRODUCTION

Allergen immunotherapy (AIT) is an effective treatment for allergic rhinitis (AR) and allergic asthma (AA), which involves the repeated administration of an allergen extract. An important question is whether AIT provides a sustained clinical effect after treatment cessation. Clinical studies suggest that a minimum of 3 years of AIT treatment results in sustainable clinical benefit and immunological changes with allergen‐specific tolerance.^1^, ^2^ AIT guidelines recommend 3–5 years of treatment, and it seems that the number of years of AIT treatment is determinant to obtain these results.^3^ Long‐term benefit is an important consideration for the recommendation of immunotherapy over standard pharmacotherapy. In general, long‐term studies evaluate efficacy of AIT treatment within 5 years after treatment discontinuation, and longer observational periods are very scarce. Long‐term treatment efficacy has been defined by the European Academy of Allergy & Clinical Immunology (EAACI) as sustained clinical benefit that lasts for at least 1 year after immunotherapy discontinuation and short‐term treatment efficacy as the clinical benefit to the patient while they are receiving immunotherapy.^2^, ^4^

## 2 METHODS

**Figure 1:**
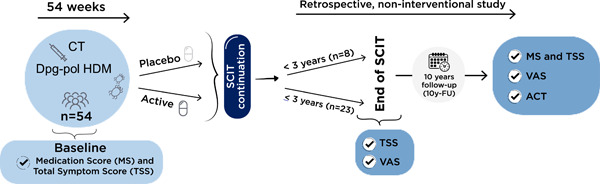
Study design.

We present data from an observational, single‐center study conducted at Hospital Universitario Ntra. Sra. de La Candelaria (Santa Cruz de Tenerife, Spain) for a 10‐year follow‐up (10y‐FU) of adult patients, who initially participated in a double‐blind placebo‐controlled clinical trial (DBPCT) and received subcutaneous immunotherapy (SCIT) treatment with a depigmented, polymerized house dust mite (dpg‐pol HDM) allergen extract or placebo during a 54 week period. Data from those patients who agreed to continue with dpg‐pol HDM SCIT (following real‐life routine clinical practice) for a further period after the completion of the DBPCT, were the objective of this study and the results are presented in this article (Figure 1).

The initial DBPCT included patients diagnosed with mild/moderate asthma with/without rhinoconjunctivitis due to HDM. Patients (*N* = 54) were divided into two groups; the active group (*N* = 27) received a depigmented‐polymerized mixture (Depigoid® 100 DPP/mL) of 50% *Dermatophagoides pteronyssinus* and 50% *Dermatophagoides farinae* (for the rest of the article referred as dpg‐pol HDM), while the control group (*N* = 27) received placebo, monthly, over 54 weeks. The results of this clinical trial showed statistically significant improvement in the active group compared with placebo control group. All the information regarding the design and results of this clinical trial can be found in a previous publication.^5^

Once the DBPCT was completed, patients from both groups were invited to receive dpg‐pol HDM under routine clinical practice. This observational retrospective study was designed to assess the long‐term effectiveness for AR and asthma of dpg‐pol HDM in a 10y‐FU visit. Changes in the Total Symptom Score (TSS) and Medication Score (MS) were the primary objectives. Visual analog scale (VAS), asthma control test (ACT), and the degree of disease control assessed by the physician were the secondary objectives. Data were analyzed at three different time‐points: baseline (before dpg‐pol HDM), end of dpg‐pol HDM SCIT, and 10y‐FU visit (Figure 1). All patients signed informed consent and this retrospective study was approved by the corresponding ethics committee.

## 3 RESULTS

Data from 31 patients were available at the 10y‐FU visit (Table 1): median age was 38 years (range 29, 53), all patients 31/31 (100%) had AA and 29/31(93.5%) had AR. Median dpg‐pol HDM treatment duration was 4 years (range 2, 5). Twenty‐three patients (74.2%) received dpg‐pol HDM for ≥3 years and 8 (25.8%) <3 years, showing a good persistence for this SCIT.

**Table 1:** Demographic data and effectiveness results of the patients included in the observational study.

	Total population (*n* = 31)	≥3 years SCIT (*n* = 23)	<3 years SCIT (*n* = 8)
*Patients' characteristics*			
Age, median (range)	38 years (32,42)	38 years (32,42)	36 years (32.5,41.5)
Sex: female (%)	19 (61.3%)	16 (69.6%)	3 (37.5%)
Rhinitis, *n* (%)	29 (93.5%)	22 (95.7%)	7 (87.5%)
Asthma, *n* (%)	31 (100%)	23 (100%)	8 (100%)
SCIT duration, median (range)* *p* = 0.0001	4 years (2,5)	5 years (4,5)*	1 year (1,1.5)*
*Endpoint*			
TSS baseline mean (SD)	24.31 (16.82)	23.76 (17.52)	25.75 (15.89)
TSS end of SCIT mean (SD)	2.16 (3.0)	1.7 (2.7)	3.5 (3.5)
TSS 10y‐FU mean (SD)	3.3 (2.9)	2.8 (2.6)	4.8 (3.3)
Change^a^ in TSS mean (SD); *p* value	−21.00 (16.56)***	−21.00 (17.60)***	−21.00 (14.54)*
MS baseline mean (SD)	18.8 (18.1)	15.5 (13.9)	28.0 (25.6)
MS 10y‐FU mean (SD)	10.4 (32.7)	3.5 (9.1)	31.1 (62.2)
Change^a^ in MS Mean (SD); *p* value	−9.3 (36.6)**	−12.8 (16.9)**	1.4 (70)
ACT 10y‐FU Median (range)	23.5 (22.0, 25.0)	23.5 (22.0, 25.0)	23.5 (20.0, 25.0)

^a^ At 10y‐FU compared with baseline.

* *p* < .05

** *p* < .01

*** *p* < .0001.

The primary objective results showed a significant reduction in TSS (mean decrease [SD] −21.00 [16.56], *p* < .0001) and MS (mean decrease [SD] −9.3 [36.6], *p* = .0003) at 10y‐FU compared with baseline. This significant reduction in TSS was also observed at end of dpg‐pol HDM treatment compared with baseline and no significant differences were observed in TSS at 10y‐FU compared with end of dpg‐pol HDM. This significant reduction in TSS was observed irrespective of treatment duration.

Only those patients who received treatment for ≥3 years experienced a significant reduction in the MS at 10y‐FU compared with baseline (mean reduction [SD] −12.8 [16.9], *p* = .0004) (Table 1). All 31 patients (100%) at baseline required inhaled steroids, however, this number was reduced to only 10 (32%) patients at 10y‐FU visit. Moreover, 10 patients (32%) did not require any type of rescue medication at 10y‐FU and all 10 had been treated for ≥3 years with dpg‐pol HDM.

**Figure 2:**
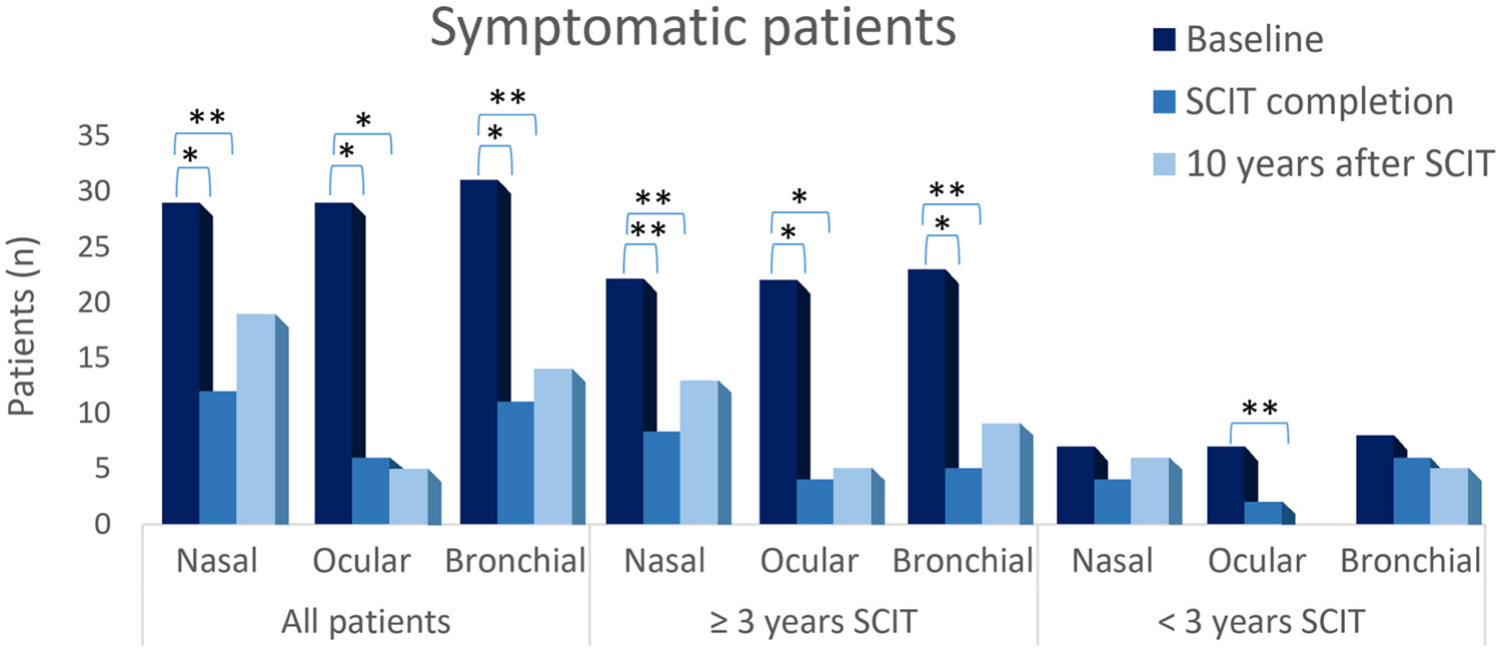
Reduction in the number of patients with allergic symptoms over time.

A significant reduction in the number of patients with specific symptoms was observed between baseline and: (a) end of dpg‐pol HDM (i) for rhinitis (*p* < .001), (ii) conjunctivitis (*p* < .0001), and (iii) asthma (*p* < .0001) and (b) 10y‐FU visit for (i) rhinitis (*p* = .0129), (ii) conjunctivitis (*p* < .0001), and (iii) asthma (*p* = .0001). However, when analyzed by treatment duration, this reduction in the number of patients with specific symptoms was only observed if treated for ≥3 years, between baseline and (a) end of dpg‐pol HDM for (i) rhinitis (*p* = .0001), (ii) conjunctivitis (*p* < .0001), and (iii) asthma (*p* < .0001), and (b) 10y‐FU for (i) rhinitis (*p* = .0117), (ii) conjunctivitis (*p* < .0001), and (iii) asthma (*p* = .0005). In the group treated <3 years, only a significant reduction in the number of patients with conjunctivitis (*p* = .0156) was observed at 10y‐FU compared with baseline (Figure 2).

**Figure 3:**
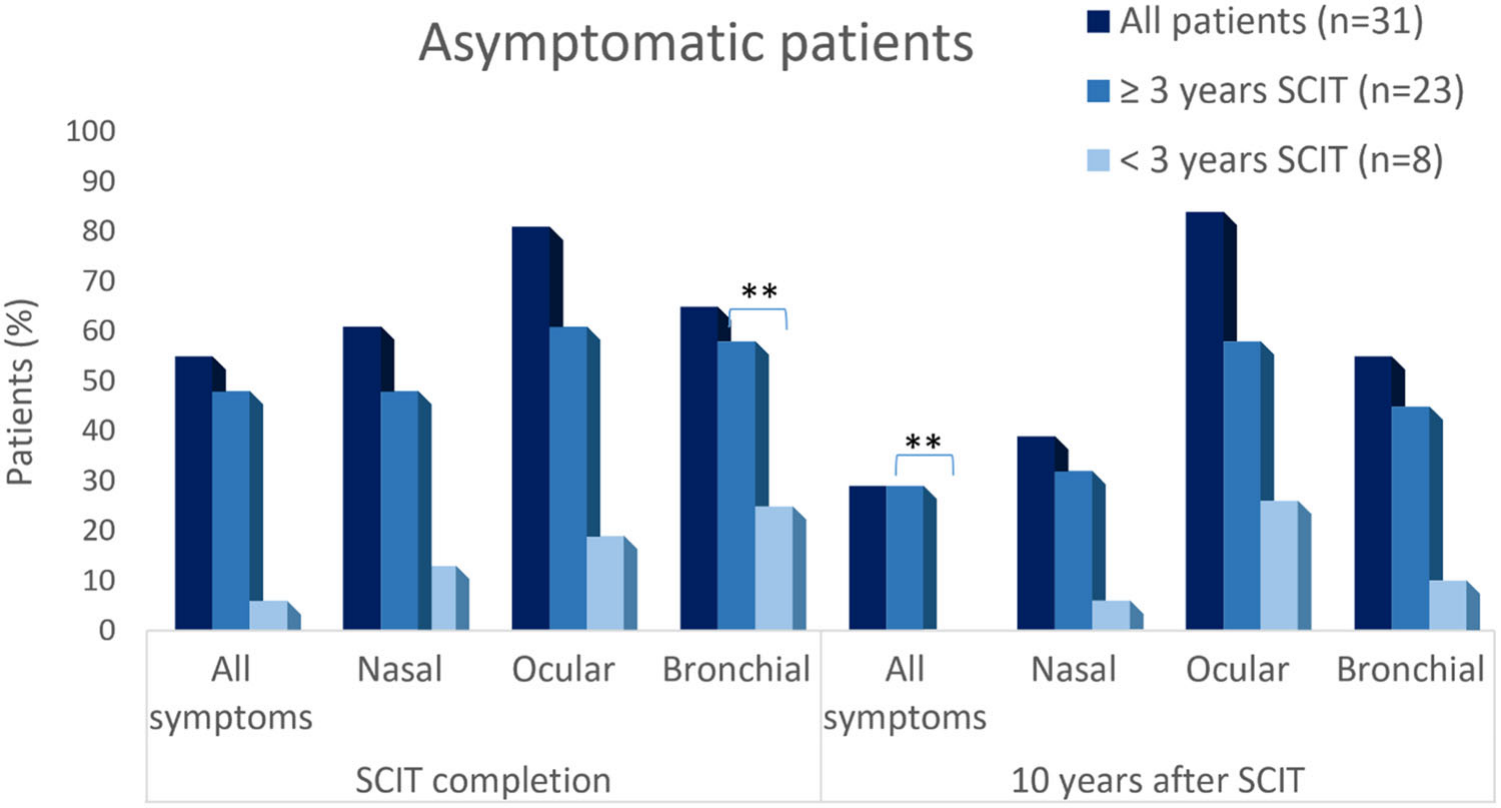
Evolution of percentage of asymptomatic patients.

Seventeen (54.8%) patients were asymptomatic at end of dpg‐pol HDM, of which 52.9% (9/17) remained asymptomatic at 10y‐FU (Figure 3). Significantly, all nine asymptomatic patients at 10y‐FU received treatment for ≥3 years (*p* = .0078).

Regarding the secondary objectives, VAS score at 10y‐FU visit assessed by patients was median 85 (range 75, 95), and likewise median 85 (range 80, 100) when assessed by investigator. No significant differences were observed between VAS at end of dpg‐pol HDM treatment compared with VAS at 10y‐FU. ACT showed a good control of asthma (ACT > 20) among allergic asthmatic patients at 10y‐FU; median 23.5 (range 22, 25) with no differences depending on dpg‐pol HDM treatment duration (Table 1). The degree of disease control was assessed by physicians and considered it controlled in 19/31 (61.3%) patients at 10y‐FU, of which 16 were treated with dpg‐pol HDM for ≥3 years and 3 for <3 years.

No adverse reactions were reported during the study period.

## 4 CONCLUSIONS

Several clinical trials and observational studies have proven that SCIT with Depigoid® HDM extract is safe and efficacious,^6^, ^7^, ^8^, ^9^ but little data are available regarding long‐term effectiveness^3^ and asthmatic's response. Recent data regarding real‐world retrospective studies have shown a reduction in the prescription of treatments up to 6 years after treatment.^10^, ^11^ Data from studies using pollen SCIT The results from this study prove that Depigoid® HDM is an effective treatment for AA and AR not only at the end of SCIT treatment, but also 10 years after AIT termination. Rhinitis, conjunctivitis and, more importantly, asthma symptoms and MS remain significantly favorable compared with baseline time‐point between the end of dpg‐pol HDM and 10 years later, demonstrating long‐term effectiveness, only when dpg‐pol HDM treatment is maintained for at least 3 years. Thirty‐nine percent and 43% of patients treated for at least 3 years were asymptomatic and did not require any rescue medication, respectively, 10 years after dpg‐pol HDM termination, reinforcing the importance of treatment duration.^3^ EAACI guidelines consider sustained long‐term clinical effectiveness that lasts at least 1 year after AIT discontinuation. In this study, we prove that with dpg‐pol HDM for at least 3 years sustained clinical effectiveness is maintained at least 10 years after AIT discontinuation. Sustained effectiveness in real life during routine clinical practice is relevant given that allergic diseases have a great socioeconomic impact. In Spain, the total mean cost per patient year of AR is 2326€^12^ and for asthma is 1726€.^13^

This study has some limitations, such as the number of patients, which is especially small when splitting between two treatment groups; however, the level of significance is very high and therefore we believe these findings are robust. Given the retrospective study design, some variables were not consistent between the initial clinical trial and the current retrospective study; however, symptom and MS, commonly used to measure efficacy in clinical trials, have been evaluated in both studies. We do believe that better‐designed long‐term prospective studies are required to confirm these findings. The lack of control group can be considered a limitation, however, ethically is difficult to avoid patients from accessing the only treatment that has proven to modify the natural course of the allergic disease for such a long period of time, to be included in a control group.

In general, these results suggest that AIT treatment duration of ≥3 years is cost‐effective as an important percent of patients remain asymptomatic and do not require rescue medication including asthma treatment, although specific studies are needed.

Given that long‐term studies (observation period after more than 5 years of AIT treatment discontinuation) are very scarce, these results help to support the international guidelines recommendation that a minimum of 3 years of AIT treatment is required for a sustained long‐term effect.

## AUTHOR CONTRIBUTIONS

Elena Rodriguez‐Plata, Ariel Callero Viera, and Jose Carlos García‐Robaina were the investigators for this study and contributed in the writing and revision of the manuscript. Aida Gomez‐Cardenosa, Eva Nieto, and Monica Ruiz‐Garcia work for LETI Pharma and worked on the statistical analysis of the results, writing and revision of the manuscript.

## CONFLICT OF INTEREST STATEMENT

Eva Nieto, Aida Gomez‐Cardenosa, and Monica Ruiz‐Garcia are employees of LETIPharma. The remaining authors declare no conflict of interest.

## ETHICS STATEMENT

Approved by the ethics committee of Hospital Universitario de La Candelaria, Tenerife, Spain. Ethics approval number: EPA‐19/15. All patients included had signed the informed consent.
